# Supplementary material for: Trends in vertical wind velocity variability reveal cloud microphysical feedback
Source: Nat Commun. 2025 Dec 22;16:11556. doi: 10.1038/s41467-025-67541-7 (PMC12749717; doi:10.1038/s41467-025-67541-7)
Supplement: Supplementary file 1 — Supplementary Information [file 41467_2025_67541_MOESM1_ESM.pdf]

Supplementary Material for Trends in Vertical Wind Velocity Variability reveal Cloud Microphysical Feedback

Donifan Barahona[1*], Katherine H. Breen[1,2], Derek Ngo[3], Flor Vanessa Maciel[3, 4], Ryan Patnaude[3, 5], Minghui Diao[3]

[1*]Global Modeling and Assimilation Office, NASA Goddard Space Flight Center, Greenbelt, MD, United States.
[2]Morgan State University, Baltimore, MD, United States.
[3]Department of Meteorology and Climate Science San Jose State University, San Jose, CA, United States.
[4]Department of Atmospheric and Oceanic Sciences, University of California, Los Angeles, CA, United States.
[5]Department of Atmospheric Science, Colorado State University, Fort Collins, CO, United States.

*Corresponding author(s). E-mail(s): donifan.o.barahona@nasa.gov; Contributing authors: katherine.h.breen@nasa.gov; derek.ngo@sjsu.edu; fvmaciel@ucla.edu; Ryan.Patnaude@colostate.edu; minghui.diao@sjsu.edu;

Keywords: Cloud microphysics, Vertical wind, Climate, Deep learning

1 Supplementary Tables and Figures

Table S1: Field campaign data used for validation. Adapted from [12].

Campaign	Year	Coverage	Flight Hours	Environment	Reference
CONTRAST	2014	20°S-40°N, 132°E-105°W	71	Tropical warm pool	[1]
HIPPO	2009-2011	67°N-87°N, 128°W-90°W	118	Pacific ocean	[2]
NSF-DC3	2012	25°N-43°N, 106°W-79°W	73	Continental United States	[3]
ORCAS	2016	75°S-18°S, 91°W-51°W	41	Southern ocean	[4]
PREDICT	2010	10°N-29°N, 87°W-38°W	92	Tropical Atlantic ocean	[5]
START08	2008	26°N-63°N, 117°W-86°W	55	North America	[6]
TORERO	2012	42°S-14°N, 105°W-70°W	54	Tropical eastern pacific	[7]
ATTREX	2014	12°S-36°N, 134°E-117°W	128	Western Pacific tropical tropopause	[8]
MACPEX	2011	26°N-41°N, 104°W-84°W	31	Southern United States	[9]
NASA-DC3	2012	30°N-42°N, 117°W-106°W	29	Continental United States	[3]
POSIDON	2016	1°S-15°N, 131°E-161°E	41	Western Pacific warm pool	[10]
SEAC4RS	2013	19°N-50°N, 80°W-120°W	15	South eastern United States	[11]

Table S2: Ground sites used for validation. The location of each site is depicted in Figure S1. Adapted from [17].

Site	Period	Altitude	Predominant Cloud Regime	Reference
NSA	07/2014 - 08/2021	Surface to 4 km	Polar stratocumulus	[13]
SGP	01/1997-08/2021	Surface to 4 km	Continental stratocumulus, Cirrus	[13, 14]
MAO	03/2014-12/2015	Surface to 14 km	Convective	[15]
COR	04/2018-04/2019	Surface to 4 km	Orographic cumulus	[13]
ENA	10/2014-07/2021	Surface to 4 km	Marine stratocumulus	[13]
ASI	09/2016-10/2017	Surface to 4 km	Marine cumulus and stratocumulus	[13]
PGH	06/2011-03/2012	Surface to 4 km	Continental stratocumulus	[13]
TWP	12/2010-01/2015	Surface to 4 km	Convective	[13]
MAN	07/1999-12/2010	6 - 14 km	Cirrus	[14]
LIM	02/2016-03/2018	6 - 14 km	Cirrus	[16, 17]
LEI	08/2011-09/2016	6 - 14 km	Cirrus	[16, 17]

Table S3: Summary of variables and inputs used for computing uncertainty in global radiative forcing from cloud microphysical effects.

**Variable**	**Values / Description**
`Preindustrial year`	1900, 1901, 1902, 1903, 1904
`Present day year`	2015, 2016, 2017, 2018, 2019
`Reanalysis`	MERRA2, ERA5, Combined
`Vertical Level`	975, 950, 925, 900, 875 (hPa)

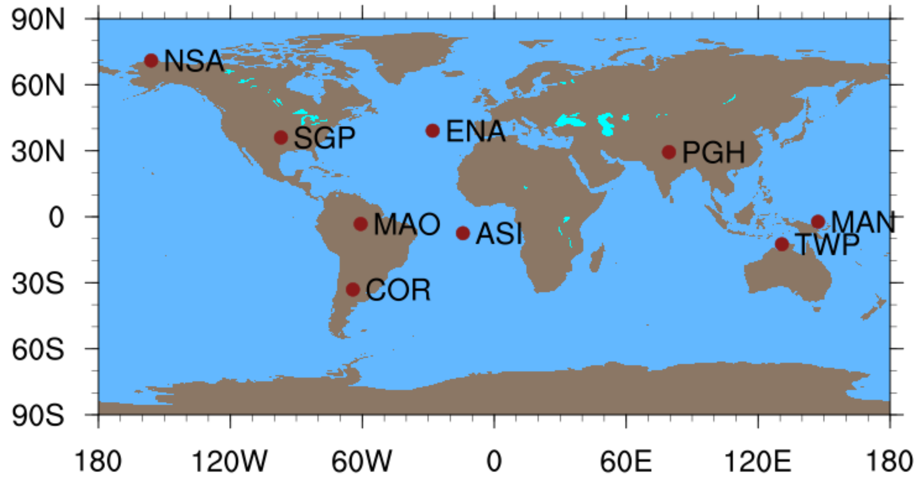

Supplementary Figure S1: Location of the ground sites described in Table S2.

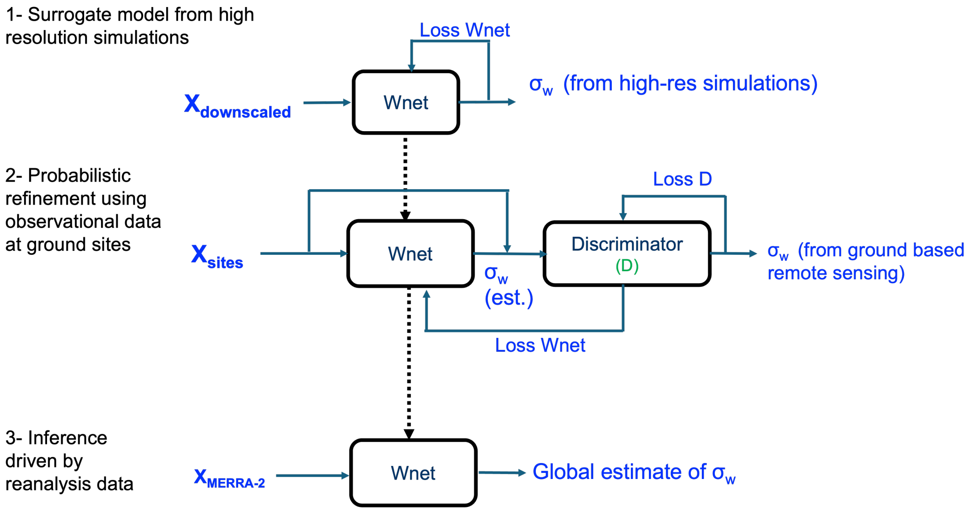

Supplementary Figure S2: Workflow for the estimation of the global distribution of σ_W. The vector $\mathbf{X}$ in each case represents the state (winds, temperature, density, water amount and coarse metrics of turbulence) obtained at the global model resolution ($\sim$ 0.5°). Steps 1 and 2 represent the development of the Wnet model detailed in reference [17]. The discriminator is an auxiliary neural network that filters out experimental error and guides Wnet to encode the true statistics of σ_W. Step 3 is performed in this work.

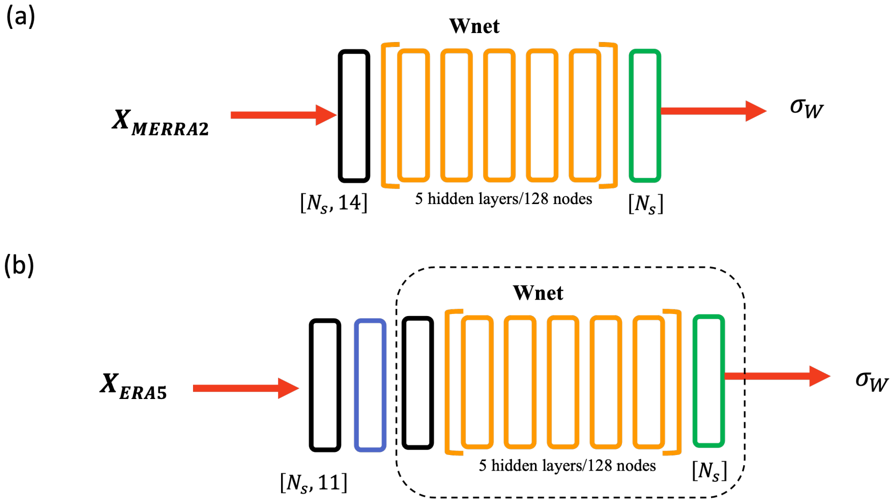

Supplementary Figure S3: Adaptation of Wnet as a foundational model for subgrid-scale variability to estimate σ_W from ERA5 data. (a) The original Wnet architecture, where black, yellow, and green represent the input, hidden, and output layers, respectively. (b) The modified architecture used to estimate σ_W from ERA5 data. Blue represents the transformational layer added to translate the input state. The dotted box indicates that the Wnet layers remain frozen during the training of the transformational layer. Here, N_s denotes the number of samples.

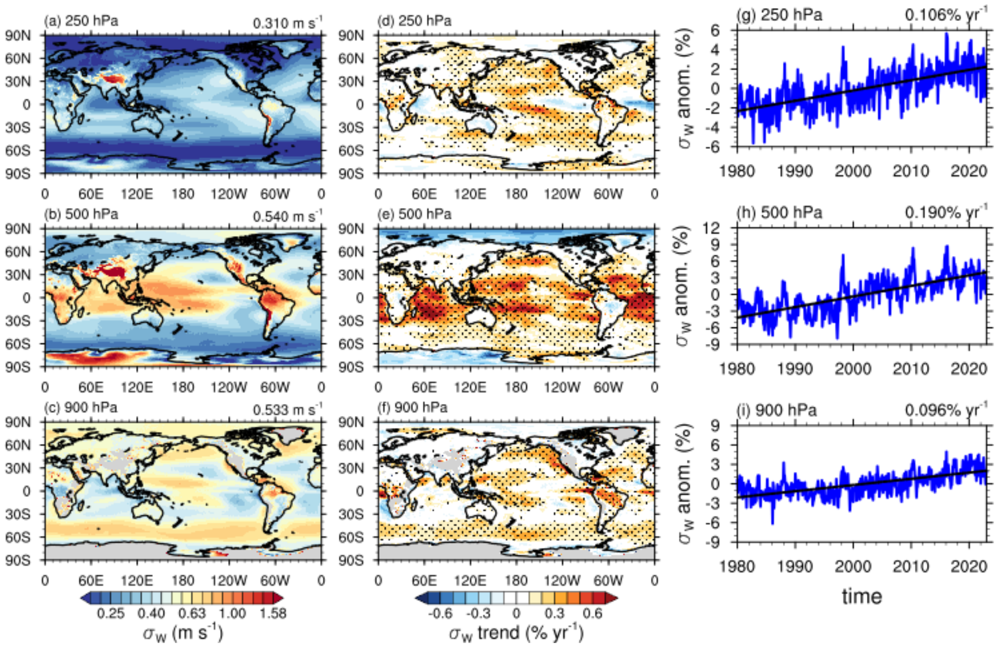

Supplementary Figure S4: Global trends in σ_W. Panels (a-c) show the annual mean σ_W for the period 1980 – 2022 computed from the MERRA-2 reanalysis on 3 h intervals. Panels (d-f) show the deseasonalized linear trend, relative to the long-term mean, derived from monthly means. Stippling highlights statistical significance at the 95% level. Panels (g-i) show the evolution of global average trend at each level. The long-term mean is displayed at the top right corner.

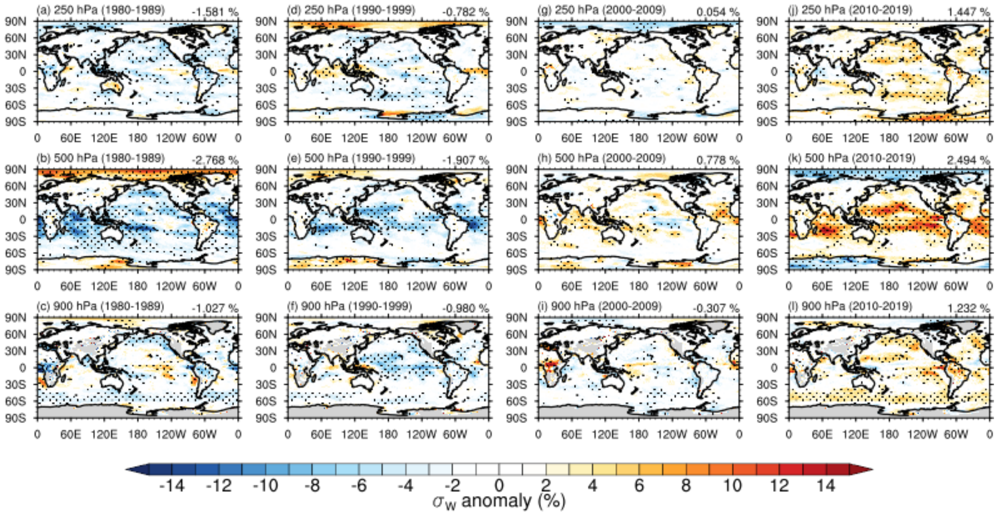

Supplementary Figure S5: Decadal mean anomaly in σ_W for different vertical levels derived from the MERRA-2 reanalysis. Stippling highlights statistical significance at the 95% level.

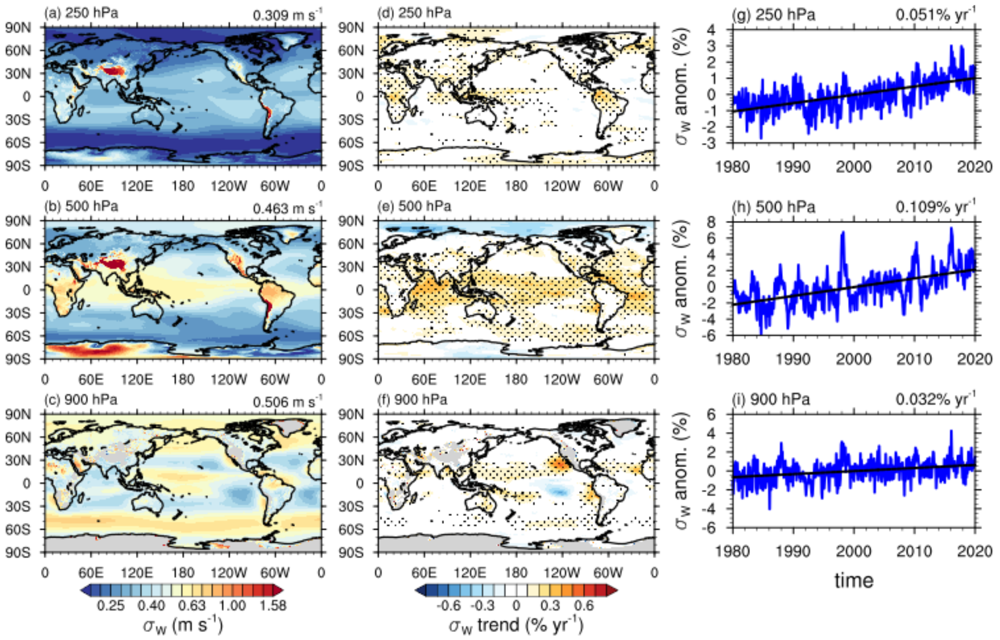

Supplementary Figure S6: Global trends in σ_W. Panels (a-c) show the annual mean σ_W for the period $1980-2022$ computed from the ERA5 reanalysis. Panels (d-f) show the deseasonalized linear trend, relative to the long-term mean. Stippling highlights statistical significance the 95% level. Panels (g-i) show the evolution of global average trend at each level. The long-term mean is displayed at the top right corner.

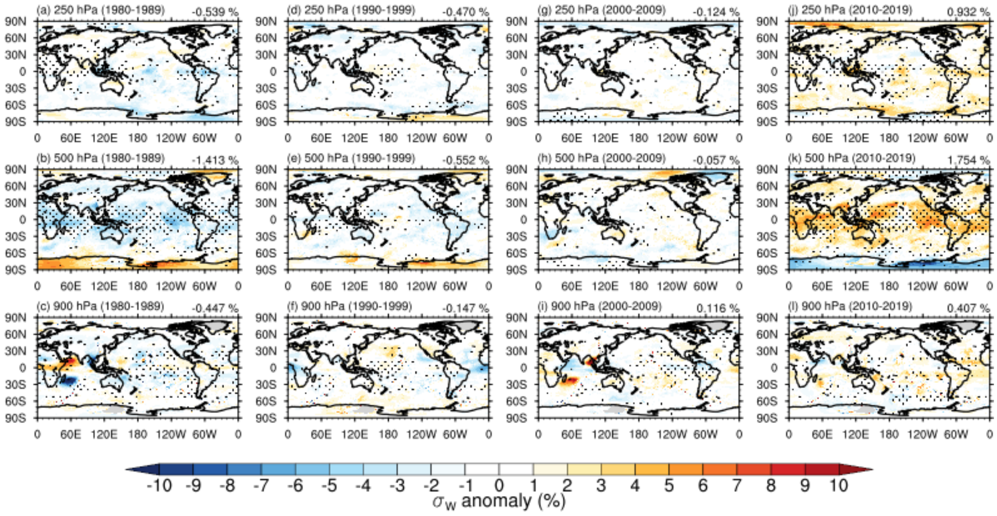

Supplementary Figure S7: Decadal mean anomaly in σ_W for different vertical levels derived from the ERA5 reanalysis. Stippling highlights statistical significance the 95% level.

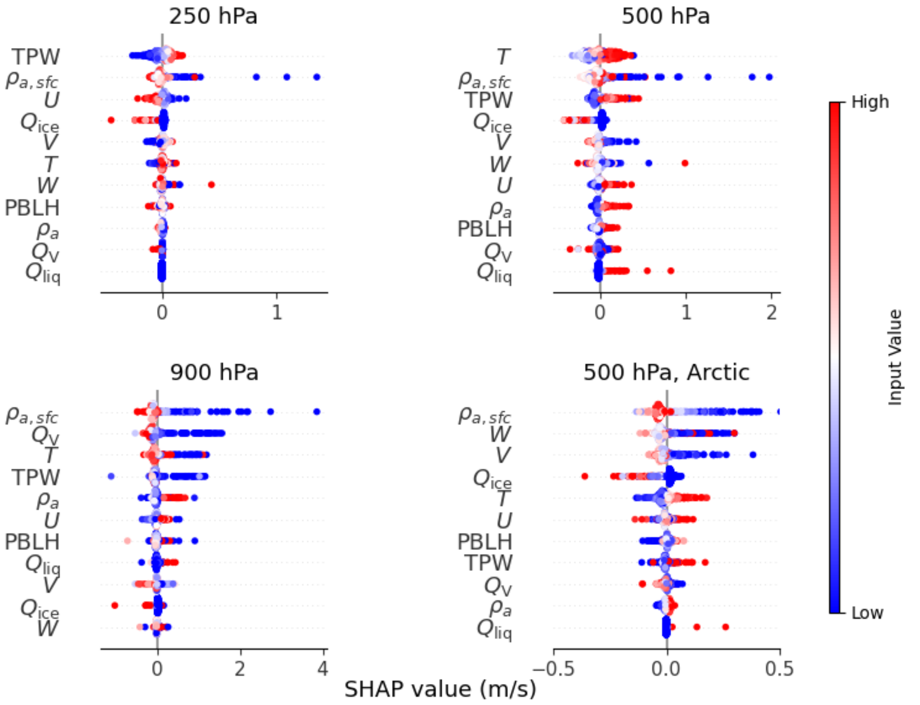

Supplementary Figure S8: SHAP values computed for σ_W [18]. These values represent the effect of each input on the overall deviation in σ_W from its base value. Each dot is a instance of the sampled values. All inputs were standardized as described in reference [17]. Red dots to the positive side of the SHAP axis indicate that increasing the input causes a positive deviation from the average (vice versa for blue dots). Inputs to the Wnet model include the planetary boundary layer height ($PBLH$, in m), total integrated water vapor (TPW, in kg m^{-2}), 3-dimensional wind velocity components (U, V, W, in m s^{-1}), water vapor, liquid, and ice mass mixing ratios ($Q_{\mathrm{v}}, Q_{\mathrm{liq}}, Q_{\mathrm{ice}}$, in kg kg^{-1}), air density (ρ_a, in kg m^{-3}), and air temperature (T, in K).

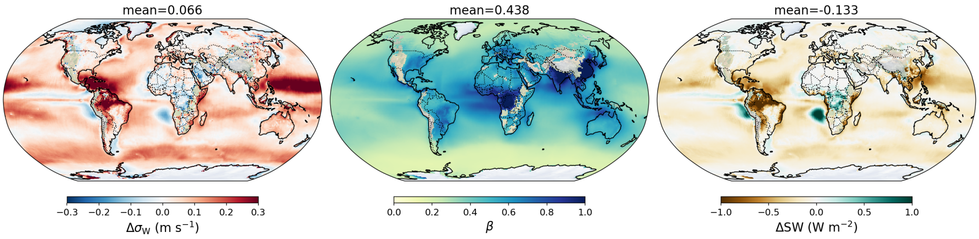

Supplementary Figure S9: Change in σ_W using the MERRA-2 reanalysis over the period 1900-2020 ($\Delta\sigma_W$), droplet number concentration susceptibility to σ_W (β), and associated shortwave radiative forcing (ΔSW).

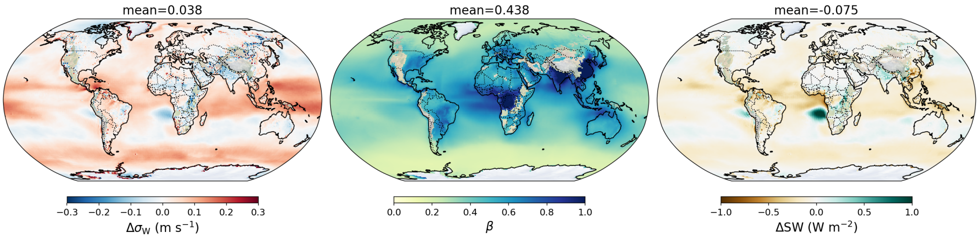

Supplementary Figure S10: Change in σ_W using the ERA5 reanalysis over the period 1900-2020 ($\Delta\sigma_W$), droplet number concentration susceptibility to σ_W (β), and associated shortwave radiative forcing (ΔSW).

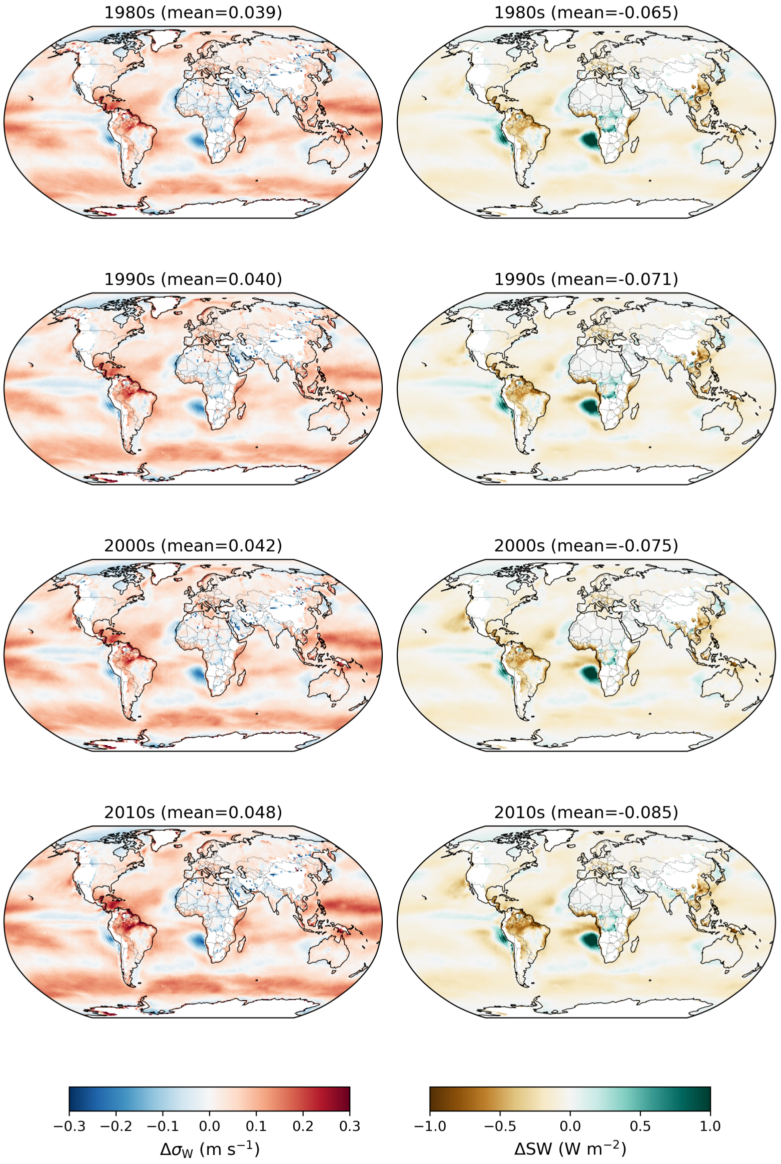

Supplementary Figure S11: Change in σ_W using the ERA5 reanalysis averaged for different decades against the 1900-1905 average ($\Delta\sigma_W$), and associated shortwave radiative forcing (ΔSW).

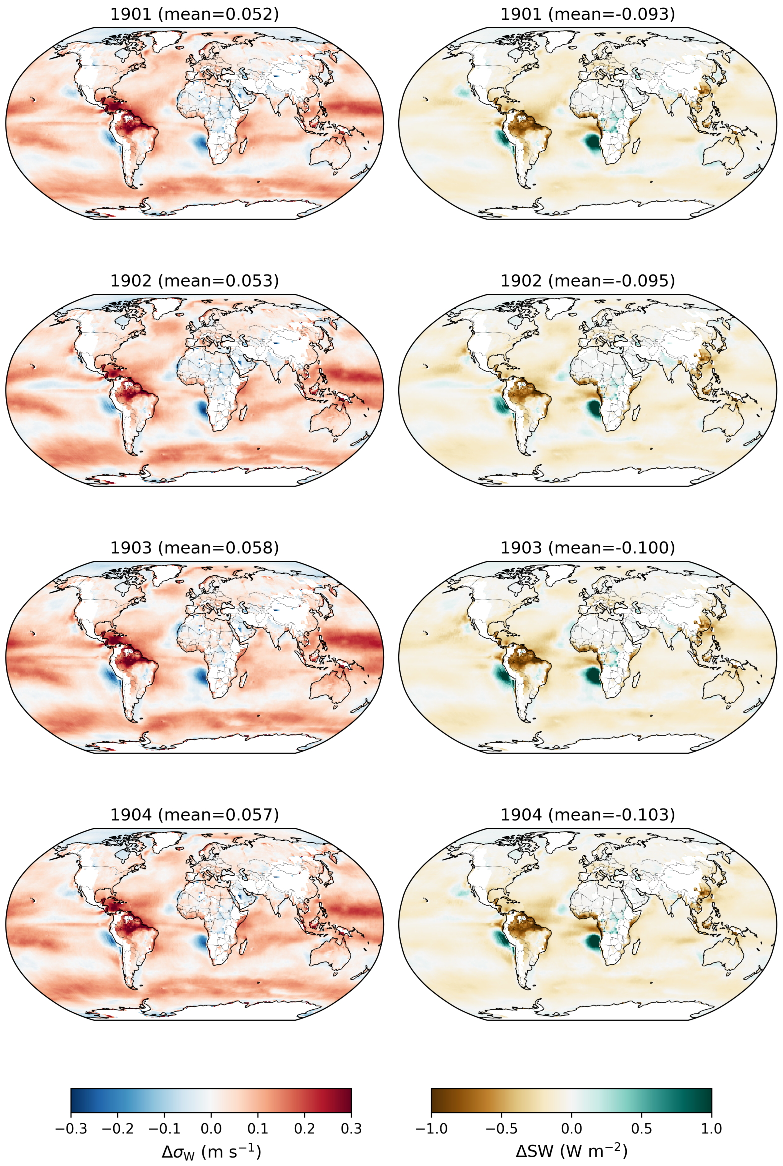

Supplementary Figure S12: Change in σ_W using the ERA5 reanalysis averaged for the years 2015-2020 against different "beginning of the century" years (1900-1905) ($\Delta\sigma_W$), and associated shortwave radiative forcing (ΔSW).

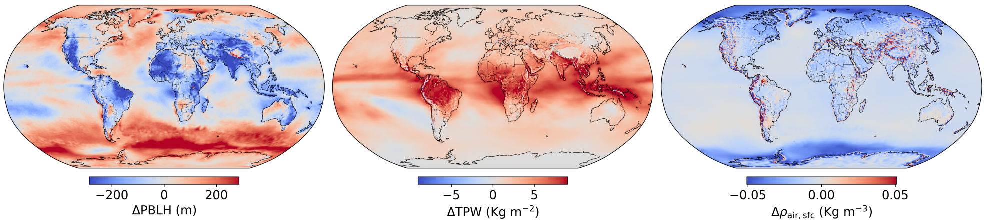

Supplementary Figure S13: Change in planetary boundary layer height (ΔPBLH), total precipitable water (ΔTPW) and surface density ($\Delta\rho_{\mathrm{air,sfc}}$) over the period 1900-2020.

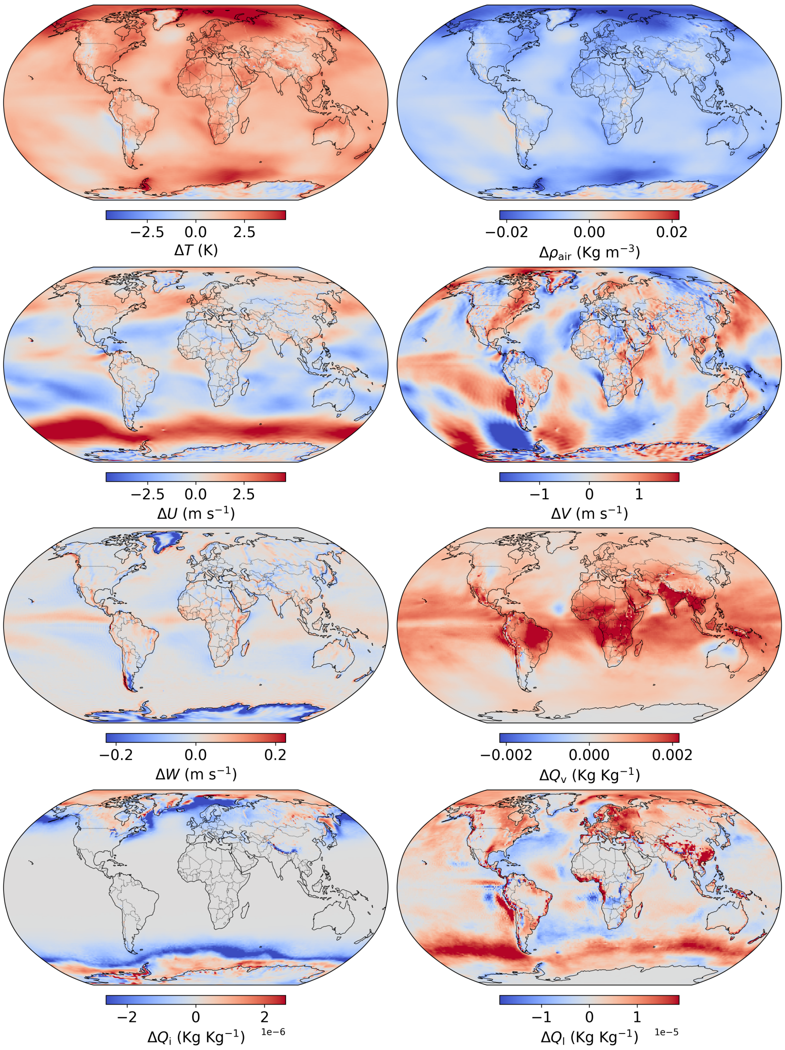

Supplementary Figure S14: Change in air temperature (T), air density (ρ_{air}), 3-dimensional wind velocity components (U, V, W, water vapor, liquid, and ice mass mixing ratios, ($Q_{\mathrm{v}}, Q_{\mathrm{liq}}, Q_{\mathrm{ice}}$), over the period 1900-2020, averaged between 875 and 975 hPa.

Supplementary References

[1] Pan, L.L., Atlas, E.L., Salawitch, R., Honomichl, S., Bresch, J., Randel, W., Apel, E., Hornbrook, R.S., Weinheimer, A., Anderson, D., *et al.*: The convective transport of active species in the tropics (contrast) experiment. Bulletin of the American Meteorological Society **98**(1), 106–128 (2017) https://doi.org/10.1175/BAMS-D-14-00272.1

[2] Wofsy, S.C.: Hiaper pole-to-pole observations (hippo): fine-grained, global-scale measurements of climatically important atmospheric gases and aerosols. Philosophical Transactions of the Royal Society A: Mathematical, Physical and Engineering Sciences **369**(1943), 2073–2086 (2011) https://doi.org/10.1098/rsta.2010.0313

[3] Barth, M.C., Cantrell, C.A., Brune, W.H., Rutledge, S.A., Crawford, J.H., Huntrieser, H., Carey, L.D., MacGorman, D., Weisman, M., Pickering, K.E., *et al.*: The deep convective clouds and chemistry (dc3) field campaign. Bulletin of the American Meteorological Society **96**(8), 1281–1309 (2015) https://doi.org/10.1175/BAMS-D-13-00290.1

[4] Stephens, B.B., Long, M.C., Keeling, R.F., Kort, E.A., Sweeney, C., Apel, E.C., Atlas, E.L., Beaton, S., Bent, J.D., Blake, N.J., *et al.*: The o2/n2 ratio and co2 airborne southern ocean study. Bulletin of the American Meteorological Society **99**(2), 381–402 (2018) https://doi.org/10.1175/BAMS-D-16-0206.1

[5] Montgomery, M.T., Davis, C., Dunkerton, T., Wang, Z., Velden, C., Torn, R., Majumdar, S.J., Zhang, F., Smith, R.K., Bosart, L., *et al.*: The pre-depression investigation of cloud-systems in the tropics (predict) experiment: Scientific basis, new analysis tools, and some first results. Bulletin of the American Meteorological Society **93**(2), 153–172 (2012) https://doi.org/10.1175/BAMS-D-11-00046.1

[6] Pan, L.L., Bowman, K.P., Atlas, E.L., Wofsy, S.C., Zhang, F., Bresch, J.F., Ridley, B.A., Pittman, J.V., Homeyer, C.R., Romashkin, P., *et al.*: The stratosphere–troposphere analyses of regional transport 2008 experiment. Bulletin of the American Meteorological Society **91**(3), 327–342 (2010) https://doi.org/10.1175/2009BAMS2865.1

[7] Volkamer, R., Baidar, S., Campos, T.L., Coburn, S., DiGangi, J.P., Dix, B., Eloranta, E.W., Koenig, T.K., Morley, B., Ortega, I., *et al.*: Aircraft measurements of bro, io, glyoxal, no 2, h 2 o, o 2–o 2 and aerosol extinction profiles in the tropics: Comparison with aircraft-/ship-based in situ and lidar measurements. Atmospheric Measurement Techniques **8**(5), 2121–2148 (2015) https://doi.org/10.5194/amt-8-2121-2015

[8] Jensen, E.J., Pfister, L., Jordan, D.E., Bui, T.V., Ueyama, R., Singh, H.B., Thornberry, T.D., Rollins, A.W., Gao, R.-S., Fahey, D.W., *et al.*: The nasa airborne tropical tropopause experiment: High-altitude aircraft measurements in the

tropical western pacific. Bulletin of the American Meteorological Society **98**(1), 129–143 (2017) https://doi.org/10.1175/BAMS-D-14-00263.1

[9] Jensen, E., Lawson, R., Bergman, J., Pfister, L., Bui, T., Schmitt, C.: Physical processes controlling ice concentrations in synoptically forced, midlatitude cirrus. Journal of Geophysical Research: Atmospheres **118**(11), 5348–5360 (2013) https://doi.org/10.1002/jgrd.50421

[10] Rollins, A.W., Thornberry, T.D., Atlas, E., Navarro, M., Schauffler, S., Moore, F., Elkins, J.W., Ray, E., Rosenlof, K., Aquila, V., *et al.*: So2 observations and sources in the western pacific tropical tropopause region. Journal of Geophysical Research: Atmospheres **123**(23), 13–549 (2018) https://doi.org/10.1029/2018JD029635

[11] Schmid, B., Flynn, C.: Studies of emissions and atmospheric composition, clouds, and climate coupling by regional surveys (seac4rs) field campaign report. Technical report, DOE ARM Climate Research Facility, Washington, DC (United States) (2016). https://doi.org/10.2172/1248492

[12] Maciel, F.V., Diao, M., Patnaude, R.: Examination of aerosol indirect effects during cirrus cloud evolution. Atmospheric Chemistry and Physics **23**(2), 1103–1129 (2023) https://doi.org/10.5194/acp-23-1103-2023

[13] Newsom, R., Sivaraman, C., Shippert, T., Riihimaki, L.: Doppler Lidar vertical velocity statistics value-added product. Technical report, DOE ARM Climate Research Facility, Washington, DC (United States) (2019). https://doi.org/10.2172/1238068

[14] Kalesse, H., Kollias, P.: Climatology of high cloud dynamics using profiling ARM Doppler radar observations. Journal of climate **26**(17), 6340–6359 (2013) https://doi.org/10.1175/JCLI-D-12-00695.1

[15] Giangrande, S.E., Toto, T., Jensen, M.P., Bartholomew, M.J., Feng, Z., Protat, A., Williams, C.R., Schumacher, C., Machado, L.: Convective cloud vertical velocity and mass-flux characteristics from radar wind profiler observations during GoAmazon2014/5. Journal of Geophysical Research: Atmospheres **121**(21), 12–891 (2016) https://doi.org/10.1002/2016JD025303

[16] Röttenbacher, J..: Further development of an algorithm to determine cirrus cloud dynamics. Master's thesis (2020)

[17] Barahona, D., Breen, K.H., Kalesse-Los, H., Röttenbacher, J.: Deep learning parameterization of vertical wind velocity variability via constrained adversarial training. Artificial Intelligence for the Earth Systems **3**(1), 230025 (2024) https://doi.org/10.1175/AIES-D-23-0025.1

[18] Lundberg, S., Lee, S.-I.: A Unified Approach to Interpreting Model Predictions (2017). https://arxiv.org/abs/1705.07874
